# The Development and Implementation of New Recommendations for Perioperative Antibiotic Prophylaxis Duration in Elective Primary Hip and Knee Replacement Surgeries

**DOI:** 10.3390/jcm15103718

**Published:** 2026-05-12

**Authors:** Nina Gorišek Miksić, Zmago Krajnc, Igor Novak, Samo Karel Fokter, Jakob Naranđa, Luka Moličnik, Andrej Moličnik

**Affiliations:** 1 Department for Infectious Diseases and Febrile Conditions, University Clinical Centre Maribor, Ljubljanska 5, 2000 Maribor, Slovenia; 2 Department for Orthopaedic Surgery, University Clinical Centre Maribor, Ljubljanska 5, 2000 Maribor, Slovenia; zmago.krajnc@gmail.com (Z.K.); igor.novak@ukc-mb.si (I.N.); samo.fokter@ukc-mb.si (S.K.F.); jakob.narandza@ukc-mb.si (J.N.); andrej.molicnik@ukc-mb.si (A.M.); 3 Medical Faculty Ljubljana, University of Ljubljana, Vrazov trg 2, 1000 Ljubljana, Slovenia; lm8339@student.uni-lj.si

**Keywords:** perioperative antimicrobial prophylaxis, surgery, implementation, barriers, facilitators, antimicrobial consumption

## Abstract

**Background**: Perioperative antibiotic prophylaxis (PAP) is effective for infection prevention in implant-related surgery, with infections being the most feared complications. A total of 15% of all antibiotics in hospitals are used for surgical prophylaxis but less than 50% of them are used according to the guidelines. International guidelines recommend only a single preoperative dose for all surgical procedures. We have developed and implemented new recommendations for PAP duration in primary hip and knee arthroplasty at the University Department for Orthopaedic Surgery. **Methods**: The development and implementation of new recommendations regarding PAP duration were performed via the following steps: pre-interventional analysis; identification of barriers and facilitators using the Flottorp framework; analyzing the data and preparation of a tailored implementation strategy based on an educational group meeting with the development of new consented to recommendations; and dissemination; followed by postinterventional analysis of PAP duration compliance 6 months later. **Results**: Before the intervention, 70% of PAP was used inappropriately (longer than 24 h). The major recognized barriers were fear of prosthetic joint infection (PJI) and a lack of concern regarding global antimicrobial resistance problems. Major facilitators were a low local PJI incidence rate (0.28%), etiology of PJI and existing local experience with a single-dose regime. After implementation of new recommendations regarding the duration of PAP, the postinterventional analysis showed that 80% of PAP was used according to the new recommendations, with a significant reduction in prolonged PAP use (from 70% to 12%), leading to an important decline in antimicrobial consumption. **Conclusions**: Our study showed that a tailored strategy in the development and implementation of new recommendations is complex and time consuming, although necessary for successful clinical practice change.

## 1. Introduction

Due to the ageing and longevity of the population, joint arthroplasties are one of the greatest medical advances for people living with severe joint pain and movement difficulties [1]. With an increasing number of procedures being performed, the number of complications is increasing as well; among them are both medical and surgical complications, which can be reasons for readmission soon after surgery. Among the most feared complications are a deep-seated surgical-site infection (SSI) or prosthetic joint infection (PJI). A PJI is now the major indication for revision surgery and is the most serious complication [2,3]. Infectious complications may result in severe joint dysfunction and an increased risk of mortality; therefore, this is the most feared complication of arthroplasty [4]. Although there have been major improvements in surgical techniques, asepsis and infection prevention, the relatively stable incidence in the last 40 years is probably the consequence of increased patient morbidity and risk factors for infection [5]. Perioperative antimicrobial prophylaxis (PAP) in arthroplasty is effective in reducing SSIs in up to 80% of cases and is a standard of care [6]. However, PAP is rarely prescribed in accordance with the recommendations, and one of the hospital antimicrobial stewardship cornerstones is the appropriate prescribing of PAP [7]. The inappropriate use of PAP may induce antimicrobial resistance (AMR), which is a major global health threat recognized by the World Health Organization (WHO). AMR renders standard treatments less efficient, and it is related to higher morbidity and mortality, and higher costs [8,9]. The WHO has emphasized that the unnecessary prolongation of PAP contributes to the emergence of microbial resistance [10]. According to the research, PAP is prescribed completely appropriately in less than 50% of cases, mostly due to prolonged use [11]. In general hospitals in Europe, 15% of the total consumption of all antimicrobial agents is for PAP, with 48.3% of prophylaxis being prescribed for more than one day, though single-dose PAP usage increased from 26.7% to 32.4% from 2016/2017 to 2022/23 [12]. Until 2017, the duration of PAP was recommended to be for up to 24 h after the procedure, and in 2017, the Centers for Disease Control and Prevention (CDC) together with different surgical associations issued new guidelines [10], where only a single preoperative dose is recommended for most surgical procedures including procedures where artificial material is inserted, i.e., all joint arthroplasties. However, some controversies in the scientific world still exist. The American Association of Hip and Knee Surgeons (AAHKS) and the American Academy of Orthopedic Surgeons (AAOS) were against a single-dose PAP approach, claiming that good-quality studies of single-dose PAP in arthroplasty were lacking [13]. A recent meta-analysis of randomized trials suggests that PAP reduces severe adverse events (including SSI) up to one year after THA with no clear benefit of multiple-dosage PAP as compared to a single-dose regime, although the certainty of evidence was low [14]. There are numerous good-quality studies available confirming the effectiveness and safety of a single-dose PAP regime in arthroplasty [15,16,17] and there are ongoing randomized prospective clinical trials in Europe and USA [18,19].

Based on the international PAP guidelines and published evidence regarding the effectiveness and safety of short-term prophylaxis, we decided to update local recommendations regarding the duration of PAP in elective arthroplasty at the University Department for Orthopedic Surgery, University Clinical Centre, Maribor, and implement it in everyday praxis. Successful implementation of new guidelines or recommendations into clinical practice is indeed very complex due to many factors influencing the process on different levels [20]. Research suggests that, on average, it takes up to 17 years for only 14% of published evidence to translate into practice [21,22]. The concept of implementation is relatively new in clinical settings. For successful implementation of changes in clinical practice, it is important to identify determinants of clinical practice: barriers and facilitators that enable or hinder the process [23]. Developing a successful implementation strategy is a complex task and selecting the right implementation strategy in different environments, different settings and different contexts is challenging [24,25]. Different frameworks were developed to analyze the determinants [26]. Flottorp et al. developed a checklist of determinants of clinical practice based on the systematic review process of 12 existing frameworks and consensus processes among implementation scientists (also known as the TICD checklist—tailored implementation for chronic diseases) [27]. This TICD checklist consists of 57 possible determinants of practice grouped into seven different main domains. Based on the recognized factors (determinants), an implementation strategy can be developed [27,28]. The TICD checklist was applied across five different chronic conditions in five European healthcare systems as part of the TICD project (Cardiovascular disease in the Netherlands, Obesity in England, Depression in Norway, COPD in Poland and multimorbidity in Germany) [29]. It was applied in primary care interventions as well as at the emergency departments for acute stroke thrombolysis implementation [30,31].

The aim of our study was to develop and implement new recommendations for PAP duration in primary hip and knee arthroplasty using the TICD checklist in the implementation process and to measure the success of the selected implementation strategy.

## 2. Materials and Methods

Based on new international guidelines for PAP and published evidence regarding the effectiveness of short-term prophylaxis, we decided to prepare and update the local recommendations regarding PAP duration in primary hip and knee joint replacement surgeries at the University Department for Orthopedic Surgery, UKC, Maribor, with the aim of shortening the duration of PAP, as recommended by the CDC. Current local recommendations recommend up to 24 h of PAP (cefazolin or vancomycin in case of allergy or MRSA colonization) for primary hip and knee arthroplasty. For successful implementation of the updated recommendation, we decided to use the Flottorp framework of determinants (TICD checklist) in the process of developing the implementation strategy [27]. The process of a new recommendation, preparation and implementation were planned in the following steps:

1. First step: pre-interventional analysis of the current praxis in PAP duration at the University Department for Orthopedic Surgery—retrospective analysis of PAP duration in 100 consecutive procedures of primary hip and knee arthroplasty.
2. Second step: initial assessment and investigation of determinants of clinical practice using the Flottorp framework (TICD checklist). Analysis of determinants was performed by conducting individual face-to-face semi-structured interviews with in-depth discussion between an infectious diseases specialist and five orthopedic surgeons performing arthroplastic surgery.
3. Third step: detailed analysis of detected determinants from interviews and from obtained existing data, and prioritizing the impact of different factors (barriers and facilitators). For overcoming recognized barriers, additional data from the Register of Endoprosthetics of Slovenia (RES) [32] regarding the local incidence of PJI after primary hip and knee arthroplasty and regarding the local etiology of PJI were obtained.
4. Fourth step: Based on all of the obtained data, an implementation strategy was developed. The implementation strategy was based on a group educational meeting with brainstorming, followed by development and implementation of new PAP recommendations. The educational group meeting was held with the surgeons and nurses at the Department of Orthopaedic surgery and it focused on the clear and accurate presentation of scientific data, presentation of local epidemiological data, presentation of detected determinants of clinical practice, and open discussion regarding barriers with local and international evidence data. Consented new recommendations were developed at the educational meeting.
5. Fifth step: dissemination of new recommendations for PAP duration in primary hip and knee arthroplasty to all department members during the morning meetings.
6. Sixth step: evaluation of the success of the implementation process by observing compliance with the new recommendation of duration of PAP in 100 consecutive hip and knee arthroplasties, 6 months after implementation.

## 3. Results

Pre-interventional analysis of the current praxis of duration of PAP in people undergoing primary hip and knee surgery at the Orthopaedic Department was performed retrospectively, observing the medical records of 100 consecutive patients undergoing elected procedures 6 months before the implementation of the new recommendations. All patients received cefazolin as prophylaxis. Only the duration of PAP (number of doses) per procedure was observed. The results are presented in Table 1.

In the pre-intervention analysis, we observed that a single-dose PAP regime was already used in some procedures (16%), while in most procedures, the duration of PAP was longer than 24 h (70%). After the initial evaluation of current practice, the infectious diseases specialist conducted individual face-to-face semi-structured interviews with all five orthopedic surgeons performing hip and knee arthroplasty at the University Department for Orthopedic Surgery, UKC. For the interviews, the Flottorp checklist (TICD checklist) for identification of barriers and facilitators in different dimensions of work was used. The seven main domains from the Flottorp checklist are as follows: guidelines factors; individual health professional factors; patient factors; professional interactions; incentives and resources; capacity of organizational change; social, political and legal factors. Among these, barriers were identified in five domains and facilitators in six domains. Patients were not included in the study. During the interviews, dimensions of clinical practice (facilitators and barriers) were thoroughly discussed. The interview results (important barriers and facilitators were recognized in different domains during the interviews) are summarized in Table 2. Among others, an important facilitator was discovered during the interview—one of the senior surgeons at the department has already been using a single dose of PAP for the last 5 years (that was also detected in the pre-interventional analysis) due to personal experience with a single-dose regime gained while visiting a foreign University hospital in 2019. Another important finding during the interviews was the misinterpretation of recommendations of 24 h of PAP by healthcare workers as administration of the last dose of PAP at 24 h after the procedure, leading to 32 h of PAP in the case of cefazolin prophylaxis (and 36 h in the case of vancomycin) resulting in 70% PAP with a duration of longer than 24 h. After the interviews and later after the detailed analysis of determinants, the determinants were prioritized in the selected implementation strategy—the fear of a PJI and lack of concern regarding rising AMR were the two most important barriers to implementation. At the same time, important facilitators were recognized; the team have a lot of knowledge regarding infection prevention in surgery as well as a lot of experience in the treatment of PJI and are very interested in improving all aspects of infection prevention.

To overcome barriers, additional data were obtained regarding the local incidence of PJI at the department in a 4-year period (2019–2023) from The Register of Endoprosthetics of Slovenia (RES) [32]. Since one of the surgeons had already been using the single-dose approach since 2019, we decided to present the PJI incidence rate by different surgeons at the department; the data regarding the surgeons were blinded. The results are shown in Table 3.

Among the five cases of PJI during the 4-year period, three were caused by Gram-negative bacteria (*Pseudomonas aeruginosa*, *Enterobacter cloacae complex*, carbapenem-resistant *Klebsiella aerogenes*) that were not susceptible to cefazolin used as PAP. No statistically significant difference in the incidence of PJI among surgeons was observed (*p* > 0.05).

After a detailed analysis of the barriers and facilitators and obtaining additional data, an implementation strategy was selected—an educational expert group meeting with the development of new recommendations in collaboration with team members. The meeting was organized with all attending surgeons, the head nurse and other members of the team. During the meeting, all of the above-mentioned data were presented together with the emphasis on the importance of PAP for the prevention of PJI, as well as the importance of other measures for SSI prevention (before, during and after surgery) that directly impact the incidence of PJI, to address the fear of a rising incidence of PJI with a shorter duration of PAP. The impact of rising AMR for the future of medicine and safe surgery was highlighted as well. International recommendations for SSI prevention were presented as well as other scientific data supporting the single-dose PAP regime, but controversies in the area were presented as well. Among the recognized important barriers was also the lack of updated national recommendation for surgical prophylaxis in orthopedic surgery and the current disagreement with single-dose prophylaxis from two major orthopedic associations in the USA (AAHKS and AAOS). The obtained data regarding the status of PAP duration at the University Department for Orthopaedic Surgery were presented, followed by local PJI incidence and etiology data after primary hip and knee arthroplasties in the 4-year period (2019–2023). Since the main recognized barrier in the implementation of a shorter duration of PAP was the fear of PJI, the information regarding the local incidence of PJI (Table 3) and the fact that the single-dose regime was already successfully used in their own facility seemed to be an important facilitator in the meeting. A very low incidence of PJI (0.28% in the 4-year period) in primary hip and knee arthroplasties at the department was reassuring and presents good overall clinical practice in all aspects of infection prevention. The third important facilitator in addressing the fear of PJIs with a shortened PAP regime was the fact that infections in the observed period were mainly caused by bacteria that were not susceptible to antibiotics used for prophylaxis, and those could not be prevented by a longer duration of PAP. In-depth discussion with an analysis of data followed, and all barriers were addressed and discussed. A tailored approach in the development of duration guidelines was used. Based on all of the available data and determinants of clinical practice, an agreement regarding PAP duration was reached with a single-dose PAP recommendation and the possibility of an additional dose postoperatively on the discretion of the surgeon, respecting surgeons’ autonomy (tailored approach). The new recommendations regarding PAP duration are presented in Table 4.

Dissemination of new recommendations was performed through morning meetings to all the doctors, department nurses and other healthcare workers involved in the patients’ care. Six months after the implementation of the new recommendations, the analysis of PAP duration was performed on 100 consecutive procedures; all patients received cefazolin as PAP. The results are shown in Table 5.

The analysis after the implementation of new PAP duration recommendations showed that the incidence of prolonged PAP (longer than 24 h) significantly declined (from 70% to 12%, 95% confidence interval (CI) 47–69%, *p* < 0.001), the percentage of the single-dose regime did not change (from 16% to 14%, 95% CI −7.9–+11.9%, *p* > 0.05), a major increase in two-dose regime was observed (from 7% to 66%, 95% CI 48.5–69.5%, *p* < 0.05) and no difference in the three-dose regime was observe (7% and 8%). Compliance with new consented recommendations (allowing an additional postoperative dose) was 80%, where most of the changes were due to the use of two doses of PAP, whereas the percentage of the single-dose PAP regime did not change and was mainly used by the same surgeon as before. A detailed analysis of cases with noncompliant PAP (more than the recommended single PAP dose postoperatively) showed that the cause of prolonged PAP was an organizational issue, where a new resident at the ward was not familiar with the new recommendation and prolonged the prophylaxis after the procedures. We addressed this issue and realized the need for regular education with recommendation dissemination.

The total consumption of antibiotics used for PAP at the University Department for Orthopaedic Surgery has declined by more than half after the implementation of the new recommendations, which is a major step toward rational antimicrobial use and optimal patient care.

## 4. Discussion

Perioperative antimicrobial prophylaxis is one of the major components of infection prevention and is universally used in implant-related surgery. On the other hand, many other factors are equally important in infection prevention. Unnecessary prolongation of surgical antimicrobial prophylaxis is very common and is seen in more than half of all surgical procedures across European hospitals [12] and around the world. Scientific data show that prolonged PAP (>24 h) does not decrease the incidence of SSI but can lead to patient-related side effects (such as *Clostridioides difficile* infections, acute kidney injuries, drug-related side effects, etc.) and globally increase AMR [33]. Implementation of a change or introduction of new guidelines into clinical practice is a complex process, and without a detailed analysis of different factors and a tailored approach, the implementation can be unsuccessful and a time-consuming activity. The selection of an appropriate determinant framework and an appropriate implementation strategy can be challenging. Different frameworks and implementation techniques have evolved in implementation science. The TICD framework helps in selecting the most important barriers and facilitators in healthcare settings, mainly through structured or semi-structured interviews, and it was demonstrated that structured group interviews with informed stakeholders were successful in identifying important determinants of clinical practice [29]. Although the TICD was developed for tailored interventions in health care and was originally used in five randomized trials, these trial results showed little impact on clinical practice [28,34]. Nevertheless, we decided to use the TICD framework in our study since it is a very systematical tool. In our study, we were able to identify the main determinants and realized that among others, the main and the priority barrier was fear of infection. Addressing this issue was complex since it can have rational, cultural and emotional components. Knowing the low local incidence of PJI (0.28%) after primary hip and knee arthroplasty and the etiology of PJI together with existing local experience with single-dose PAP was reassuring for the surgeons, yet it was not enough to result in a complete switch to a single-dose PAP regime with the new PAP recommendations. We decided to choose persuasion based on epidemiological and scientific facts though an educational meeting as the main implementation strategy for overcoming the barrier of fear, although a pragmatic and adaptive approach in the development of recommendations was necessary. Expert consensus with the possibility of a single additional dose was reached during the meeting, since it was clear that some autonomy of the surgeon in terms of PAP duration must be maintained. Consented recommendations in this sociocultural setting is more appropriate and has led to higher compliance. According to cultural dimensions by Hofstede [35], Slovenia has a high uncertainty avoidance index (88) and a high power distance index (71), which are both related to a higher rate of antibiotic prescribing [36]. A high uncertainty avoidance index is related also to prolonged PAP (longer than 24 h) in European countries [37], referring to a cultural tendency to favor a longer duration of PAP, which is a probable reason for surgeons’ hesitation in switching to a single-dose PAP regime. Insisting on new recommendations (for single-dose PAP) without consensus would probably lead to the failure of the intervention without any or little desired improvement. Instead, tailored implementation of the new recommendations was successful with an 80% compliance rate after 6 months, with an important decline in PAP antimicrobial consumption.

Although this strategy resulted in a significant reduction in prolonged PAP use (>24 h), the recommended option of single-dose PAP was not successfully implemented, since most surgeons still decided to use a second postoperative dose.

In our study we used a simple implementation strategy based on an educational meeting with evidence-based persuasion. It is probable that additional and more complex implementation strategies should be used to achieve better results in the future. In the field of implementation science, there are many different tools with more than 60 implementation frameworks that can help in planning a successful intervention and selecting a successful strategy. Implementation mapping is a stepwise approach for developing strategies in health care based on theory and evidence-based interventions, and this helps implementers to identify change objectives and select behavioral change techniques [38]. Another tool frequently used in implementation processes is the Theoretical Domain Framework (TDF) combined with COM-B wheel (Capability, Opportunity, Motivation–Behavior) of behavioral change that can help to link domains with interventions. The TDF was developed by behavioral and implementation scientists and is based on behavioral science and behavioral change techniques [39]. The Consolidated Framework for Implementation Research (CFIR) is yet another framework and is among the most widely applied in healthcare settings since it is comprehensive and adaptable, and it can also be used in combination with other frameworks (such as the TDF; where the a CFIR provides more comprehensive set of implementation determinants and the TDF focuses on behavioral change) [40,41]. The Expert Recommendations for Implementing Change (ERIC) project was developed, among others, to standardize terminology in this field, and it offers a curated list of evidence-informed strategies organized into categories and provides a much more structured way to match a barrier with an appropriate strategy [42].

Despite numerous techniques and strategies in the implementation of changes in clinical practice, it has been proven that methods using financial penalties for non-compliance can also be highly effective, as demonstrated by Wood et al. [43]. In Slovenia, such an approach is not feasible at this time due to a very complex method of healthcare financing in the public sector, although with a fast-developing private sector in healthcare, this approach could be applicable in many different areas of health care.

A major limitation of our study is that the postinterventional observation was conducted only 6 months after implementation, which represents a short period for observing the sustainability of a clinical practice change. Another limitation of our study was not including the patients and analyzing their factors that could influence the implementation strategy of a short PAP duration. The understanding of the problem of AMR by European citizens is low according to the survey by the Kantar Public Brussels network in the 27 EU Member States in 2022, with 72% of respondents lacking some essential knowledge about antibiotics. On the other hand, 86% of them stated that doctors are the most trusted source of information regarding antibiotic use [44]; the majority of them were aware that the unnecessary use of antibiotics will make them become inefficient in the future. This knowledge should be included in the preoperative conversation between the surgeon and the patient regarding all of the risks of the surgery including the importance of PAP and other infection-prevention measures, reassuring the patient that the optimal duration of PAP means optimal care, where an SSI is not preventable by prolonging PAP. It is also important to emphasize that we will continue to actively monitor the incidence of PJIs at the department after the implementation of the modified PAP regime.

## 5. Conclusions

Consented development of new recommendations with tailored implementation addressing the barriers resulted in the successful shortening of the duration of PAP and the optimization of patient care with lower unnecessary antibiotic consumption. Knowledge of the local data regarding the current practice, incidence and local epidemiology of PJI are all important tools in overcoming barriers to implementation. For successful implementation, a multistep process is needed, where all involved stakeholders actively participate, and further activities in terms of continuous education, improvements, regular audits with feedback are needed. Although the whole process is time consuming, it is necessary for clinical practice change.

## Figures and Tables

**Table 1:** Duration of perioperative antibiotic prophylaxis with cefazolin in primary elective total hip arthroplasty and total knee arthroplasty before the implementation of new recommendations.

Duration of PAP:	Number of Procedures (N = 100, %)
Single preoperative dose	16 (16%)
1 dose postoperatively	7 (7%)
2 doses postoperatively	7 (7%)
3 doses postoperatively ^1^	70 (70%)

^1^ All 70 received 32 h of PAP; nobody received PAP for longer than 32 h.

**Table 2:** Barriers and facilitators recognized during individual interviews with surgeons (according to the TICD—tailored implementation for chronic diseases checklist).

Barriers:	Facilitators:
**1. Domain: Guideline factors:**	**1. Domain: Guideline factors:**
Quality of evidence: the quality of international recommendations has not been assessed properly by the orthopaedic team; majority of the surgeons were not aware of the level of evidence of the new CDC * guidelines.	Clarity and the strength of recommendation: CDC * guidelines do not recommend an additional postoperative dose of PAP ** (single-dose PAP ** regime); the recommendation is graded as strong (category IA)
Lack of clarity of the current local recommendations: surgeons were convinced that 24 h of PAP ** means the patient receives the last PAP ** dose 24 h after the procedure resulting in 32 h of PAP ** (>24 h).	Feasibility: single-dose PAP ** regime is easier to perform and more practical: more practical for the surgeons (no need to prescribe additional antibiotics postoperatively) and it is also time sparing for the nurses on the hospital ward.
Accessibility of recommendation: The CDC * guidelines 2017 were not priority guidelines in orthopaedic surgery.	
Local appropriateness: National recommendations on PAP in orthopaedic surgery have not been updated since 2013 and they are still recommending PAP ** for 24 h after the procedure.	Accessibility of the intervention: this intervention is easily accessible in every clinical setting.
Lack of confidence in international guidelines: The international guidelines recommending single-dose PAP ** were published by a foreign institution from the United States (CDC *).	Compatibility: The recommended PAP ** change (single-dose PAP ** regime) fits well with current practice and it does not change the current workflow. It is also interesting for the possible future “one-day surgery” where arthroplasty procedures will be performed on an outpatient basis.
Source of recommendation: The CDC * and ECDC *** are generally considered to be credible organizations, although in the field of surgical infections, they are not recognized as the most important ones.	Effort: There is not a lot of additional effort needed for the execution of this change.
Lack of consistency with other guidelines: opposite opinions from different associations (CDC *, AAOS **** and AAHKS *****).	
Observability: due to low incidence of prosthetic joint infections, a longer observational time is needed to prove noninferiority of a shorter PAP ** regime locally (local data).	
**2. Domain: Individual health professional factors:**	**2. Domain: Individual health professional factors:**
Knowledge about own practice: there were no systematic audits with feedback of PAP ** at the department, although they were aware that different PAP ** regimes are in place.	Knowledge: highly skilled surgeons and other healthcare workers at the department with a lot of pre-existing knowledge and expertise in the field of surgical-site infection and prosthetic joint infection prevention and treatment.
Agreement with single-dose recommendation: majority of surgeons were moderately reserved regarding single-dose PAP ** regime during the interview due to expected bad outcome (fear of surgical-site infection) which would lead to increased short-term stress. They perceived the proposed change in PAP ** duration (single-dose PAP ** regime) as an interference with the prescriber’s autonomy.	Awareness of new foreign recommendations: one senior surgeon at the department already uses a single-dose PAP ** regime; others were aware of this practice.
Intention and motivation: antimicrobial resistance is not surgeons’ main concern in individual patient care after arthroplasty—antimicrobial resistance is not perceived as an immediate threat in individual surgery and, consequently, they are not directly motivated to adhere to a short PAP ** regime.	Skills needed to adhere: no additional skill is needed for the single-dose protocol.
Self-monitoring and feedback: systematic prospective local monitoring with feedback in the field of PAP ** duration is lacking in our institution.	Attitudes toward guidelines: surgeons have a positive attitude towards guidelines in general and do not feel restricted by them when they agree with guidelines (and if the guidelines do not restrict them).
Emotions: emotions—especially the fear of surgical-site infections—importantly hinders the acceptance of a shorter PAP ** regime.	Intention and motivation: they intend to adhere to a change in clinical practice if they agree with the change.
	Learning style: expert meeting and personal communication is the best way to learn new things.
	Capacity to plan change: healthcare professionals have skills and experience to plan the changes needed for implementation of new recommendations
**3. Patient factors**	**3. Patient factors**
Patient knowledge: The general knowledge regarding the rising antimicrobial resistance rate in our population is poor.	Patient needs: in our cultural settings, patients will probably not have any demands regarding the duration of PAP **.
	Patient motivation: no patient motivation is needed to adhere to new PAP ** recommendation
**4. Domain: Professional interactions**	**4. Domain: Professional interactions**
No barriers were found in this domain.	Communication and influence: communication among colleagues is respectful with good team interaction between all members of the healthcare system at the department.
	Team processes: surgeons and other team members (hospital and surgical nurses) are aware of the importance of all measures regarding infection prevention in patients undergoing arthroplastic surgery.
**5. Domain: Incentives and recurses**	**5. Domain: Incentives and recurses**
Non-financial and financial incentives or disincentives: there are no financial or non-financial incentives for lowering the hospital’s antimicrobial costs and lowering antimicrobial resistance rates.	Availability of necessary resources: no additional resources are needed for this change.
Informational system: hospital informational system does not facilitate adherence to guidelines or to any recommendations (no integrated decision-support system).	
Assistance for clinicians: clinicians do not have any assistance (checklist for PAP ** duration) that would help them in adhering to new recommendations.	
**6. Domain: Capacity for organizational change**	**6. Domain: Capacity for organizational change**
Relative strength of supporters and opponents: the relative strength of individuals in this team—every surgeon’s opinion is equally important.	Mandate, authority and accountability: the head of the department has a mandate and the authority to make a process change and is motivated to improve the quality of patient care.
Priority of necessary change: this change in PAP ** recommendation is of low priority in their everyday praxis.	Capable leadership: leaders support quality improvement in all fields of patient care at our institution.
Monitoring and feedback: real-time monitoring of PAP ** duration is not available; no regular audit and feedback is available.	Regulations, rules and policies: orthopaedic expert team is independent in the implementation of new scientifically based clinical protocols.
**7. Domain: Social, political and legal factors**	**7. Domain: Social, political and legal factors**
No barriers were found in this domain.	No economic constraints hinder this new change in recommendations.

* CDC—Centers for Disease Control and Prevention. ** PAP—Perioperative antibiotic prophylaxis. *** ECDC—European Center for Disease Control. **** AAOS—American Academy of Orthopaedic Surgeons. ***** AAHKS—American Association of Hip and Knee Surgeons.

**Table 3:** Number of primary hip or knee arthroplasties per surgeon and number of prosthetic joint infections (PJIs) per surgeon in 2019–2023 period.

Surgeon	Number of Procedures	Number of PJIs (Incidence in %)
A	314	0
B	364	1
C	361	2
D	409	2
E	305	0
X *	31	0
Total	1784	5 (0.28%)

* Unknown surgeon in database; PJI—prosthetic joint infection.

**Table 4:** New recommendations for duration of perioperative antibiotic prophylaxis in primary hip and knee arthroplasty.

Antibiotic of Choice:	Duration of Perioperative Prophylaxis:
Cefazolin:	A single dose of cefazolin preoperatively is recommended, although up to maximum of 1 additional dosage postoperatively can be used if the surgeon decides to do so (1 dose preoperatively; additional dose at 8 h postoperatively).
Vancomycin *:	A single dose of vancomycin preoperatively is recommended, although up to maximum of 1 additional dose be used if the surgeon decides to do so (after 12 h).

* Vancomycin is used in patients with penicillin allergies and patients colonized with methicillin-resistant *Staphylococcus aureus* (MRSA).

**Table 5:** Duration of perioperative antibiotic prophylaxis with cefazolin six months after the implementation of new recommendations.

Duration of PAP:	Number of Procedures
Single dose	14/100
1 dose postoperatively	66/100
2 doses postoperatively *	8/100
3 doses postoperatively **	12/100

* Two doses of cefazolin postoperatively up to 24 h, ** three doses of cefazolin postoperatively are >24 h of PAP.

## Data Availability

The data that support the findings of this study are available from the corresponding author upon reasonable request.

## Abbreviations

The following abbreviations are used in this manuscript:

PAP	Perioperative antimicrobial prophylaxis
PJI	Prosthetic joint infection
SSI	Surgical-site infection
AMR	Antimicrobial resistance
WHO	World Health Organization
CDC	Centers for Disease Control and Prevention
AAHKS	American Association of Hip and Knee Surgeons
AAOS	American Academy of Orthopedic Surgeons
TICD	Tailored implementation for chronic diseases
